# Circular RNA expression profiles of peripheral blood mononuclear cells in hepatocellular carcinoma patients by sequence analysis

**DOI:** 10.1002/cam4.2010

**Published:** 2019-02-04

**Authors:** Bo Lei, Jian Zhou, Xiuyun Xuan, Zhiqiang Tian, Mengjie Zhang, Weiwu Gao, Yuxin Lin, Bing Ni, Hui Pang, Weiping Fan

**Affiliations:** ^1^ Department of Microbiology and Immunology Shanxi Medical University Taiyuan China; ^2^ Department of Pathophysiology Third Military Medical University Chongqing China; ^3^ Department of Kidney Southwest Hospital Third Military Medical University Chongqing China; ^4^ State Key Laboratory of Silkworm Genome Biology Southwest University Chongqing China; ^5^ Bellevue Christian High School Bellevue Washington; ^6^ Department of Immunology Changzhi Medical College Changzhi China

**Keywords:** biomarker, circRNAs, hepatocellular carcinoma, high‐throughput sequencing

## Abstract

Circular RNAs (circRNAs) are a large class of noncoding RNAs that have potential regulatory roles in disease pathogenesis and progression. Recently, circRNAs have been found to be expressed in hepatocellular carcinoma (HCC) tissues and involved in the development and metastasis of HCC. However, the significance of circRNAs in peripheral blood mononuclear cells (PBMCs) of HCC patients remains unclear. In this study, RNA sequencing analysis was performed to identify circRNAs from four HCC patients and three healthy controls to determine the expression pattern of circRNAs in the PBMCs and the circRNAs’ molecular regulatory networks in HCC pathogenesis. A total of 58 circRNAs were found to be significantly changed (≥2 or ≤0.5‐fold) in the PBMCs of HCC patients compared with those of the healthy cases. Six random representative circRNAs (three up‐ and three down‐regulated) were further validated by real‐time RT‐PCR in 72 samples of PBMCs from HCC patients and 30 control subjects. Chi‐square test indicated that one of the up‐regulated circRNA candidates—circ_0000798—was correlated with clinical variables. Highly expressed circ_0000798 was associated with poor overall survival of HCC patients. Receiver operating characteristic curve analysis further revealed that circ_0000798 was discriminating HCC patients from healthy controls. Finally, the predicted competing endogenous RNA network of circ_0000798 showed that it might act as a “sponge” of target microRNAs, that would subsequently regulate the expression of target genes in PBMCs. In summary, this is the first study to comprehensively identify dysregulated circRNAs in PBMCs of HCC patients, and its findings suggest that dysregulated circ_0000798 in PBMCs has potential as a convenient biomarker for diagnosing or prognosticating HCC.

## 1 INTRODUCTION

Among the most common malignancies worldwide, hepatocellular carcinoma (HCC) is the most common primary malignancy of the liver and exhibits one of the highest mortality rates.^1^ More than 750 000 people are newly diagnosed with HCC every year globally.^2^ Although advances in diagnosis, surgical techniques, and liver transplantation have been achieved, the long‐term survival of patients with HCC remains poor to date. Moreover, the prognosis after curative resection of HCC has remained unsatisfactory because of a high incidence of postoperative recurrence.^3^ Identifying early diagnostic and prognostic biomarkers for metastatic HCC is of paramount importance to overcome these ongoing challenges to human health.

Circular RNAs (circRNAs) are a type of endogenous RNA with a stable structure that is widely expressed in mammal genomes.^4^ The circRNAs were initially observed in RNA viruses in the 1970s^5^ and were considered mere by‐products of splicing in the 1990s.^6^ However, the advent of RNA sequencing technology and bioinformatics in the recent decades have led to the novel and extraordinary findings that circRNAs are abundant, conserved, and stable, have a tissue‐specific expression pattern in mammalian cells,^4^, ^7^ and are unique noncoding RNA (ncRNA) molecules, with sizes ranging from hundreds to thousands of nucleotides. Although most of the circRNAs discovered to date appear to not encode proteins, the circRNAs in general have been shown to play crucial regulatory roles in a variety of cellular processes and biological pathways,^8^ similar to other ncRNAs such as microRNAs (miRNAs) and long noncoding RNAs (lncRNAs). The regulation mechanisms implicated to date include cell proliferation, apoptosis, differentiation, and angiogenesis.^9^, ^10^ A number of studies have also shown a close relationship between circRNAs and cancers, wherein circRNAs could regulate the mRNA activity of target genes by acting as miRNA sponges, promoting or inhibiting the development and metastasis of various cancer types.^11^, ^12^, ^13^ Collectively, these lines of evidence provide a new direction for exploring circRNAs as targets for the diagnosis and prognosis of diseases.

It is well known that under normal physiologic conditions immune cells monitor and clear mutated cells from the body. However, the immune cells can also establish conditions supportive of tumor growth and invasion, such as the proinflammatory environment that develops during chronic inflammation.^14^ The peripheral blood mononuclear cells (PBMCs) are composed of various leukocyte subpopulations, including T lymphocytes, B lymphocytes, monocytes, natural killer cells, and dendritic cells. Studies of the key genes in PBMCs have found associations between altered gene expression and diseases, such as cancer, and disease states, such as cancer‐related immunity.^15^ Thus, altered gene expression in PBMCs has been proposed as a potential clinical tool, as it may reflect a disease's diagnosis or prognosis. Moreover, since, as described above, circRNAs are capable of regulating gene expression and the dysregulated gene expression in PBMCs might, at least partially, attribute to the altered circRNAs profile observed in PBMCs, the expression profile of circRNAs in PBMCs might represent a promising tool for discovering biomarkers associated with physiological or pathological events. For example, determining how the circRNAs profile is changed in PBMCs of HCC patients and whether those differential expression profiles could be useful for the diagnosis or prognosis of HCC.

In this study, we used high‐throughput sequencing to identify aberrantly expressed circRNAs in the PBMCs of HCC patients, as compared to healthy controls. The sequencing efficiency was validated by the selected dysregulated circRNAs by quantitative reverse transcription (qRT)‐PCR, and the putative circRNA‐miRNA‐mRNA interaction was predicted for the selected dysregulated circ_0000798. Finally, a receiver operating characteristic (ROC) curve was used to analyze the potential diagnostic and clinical significance of circ_0000798 for HCC.

## 2 MATERIALS AND METHODS

### 2.1 Sample collection

A total of 102 peripheral blood samples were collected during surgery (72 patients with HCC and 30 healthy controls) between 2016 and 2018 in the Southwest Hospital of the Third Military Medical University (Chongqing, China). The patients had not been treated with radiotherapy or chemotherapy before surgery. Healthy subjects who received a regular physical examination at the Department of Health from the same hospital were recruited as normal control subjects. Fresh peripheral blood samples (10 mL) were collected from all of the subjects in ethylenediaminetetraacetic acid (EDTA) tubes. This study was approved and supervised by the ethical committee of the Southwest Hospital. Written informed consent was obtained from all subjects in this study.

### 2.2 PBMC preparation and total RNA extraction

Fresh 10‐mL peripheral blood samples were collected in EDTA tubes before use in the study procedures. Within 4 hours, PBMCs from each donor were isolated using density centrifugation (700 *g* for 20 minutes) with the total 10‐mL blood sample layered on 5 mL Ficoll‐Paque PLUS (Cat. No. 17‐1440‐02; GE Healthcare, Uppsala, Sweden) at room temperature. The cells were subsequently frozen in TRIzol reagent (Invitrogen, Carlsbad, CA, USA) and stored in liquid nitrogen (−180°C). Total RNA was extracted from the isolated PMBC samples using TRIzol reagent according to the manufacturer's instruction. The quantity of the isolated RNA was measured using a NanoDrop spectrophotometer (Agilent Technologies, Santa Clara, CA, USA).

### 2.3 Whole transcriptome sequencing

The RNA extracted by TRIzol reagent and having an RNA integrity number of >8.0 was utilized to construct an rRNA depletion library (VAHTSTM Total RNA‐seq [H/M/R]; Agilent Technologies) according to the manufacturer's instructions. Whole transcriptome sequencing was performed by NovelBio Corp. Laboratory (Shanghai, China) using a Hiseq^™^ XTEN Sequencer (Illumina, San Diego, CA, USA). The sequencing data were first filtered utilizing domestic Java code (removing the adaptor sequences, reads with >5% ambiguous bases (noted as N), and low‐quality reads containing more than 20% of bases with qualities of <20) and then mapped to the human genome (human genome version: GRCH38.p7 National Center for Biotechnology Information [NCBI]) utilizing HISAT2.^16^

### 2.4 Data analysis and identification of differentially expressed circRNAs

First, low‐quality reads were removed from the raw sequence data. The Unmapped Reads Collection (HISAT2)^17^ was then used as the method of mapping to the reference genome (GRCH38.p7 NCBI; utilizing the parameter ‐hisat2 ‐p 8 ‐5 5 ‐3 5 –min‐intronlen 20‐max‐intronlen 500000 ‐k 3 –phred33.). Next, the pipeline “acfs2” (publicly available at https://code.google.com/p/acfs2/) was used to identify candidate circRNA in each sample, according to the procedures described in the published literature.^18^

### 2.5 qRT‐PCR validation of differentially expressed circRNAs

The dysregulated expressions of six randomly selected circRNAs from our RNA sequencing data were validated by qRT‐PCR. For this, cDNA was first synthesized from 1 μg total RNA using a Prime‐Script RT reagent kit with gDNA Eraser (TaKaRa, Shiga, Japan). Then, specific divergent primers for each circRNA were designed based on circPrimer1.2 (http://www.bioinf.com.cn/) and according to the “spliced sequence” of circRNA originated from our sequence data (consistent with circBase [http://www.circbase.org/]) (Table 1). The real‐time PCR was performed using SYBR qPCR Super Mix (Novoprotein, Shanghai, China) with the Stratagene Mx3000P Real‐Time PCR System (Agilent Technologies) and following the manufacturer's instructions. The PCR reactions were: denaturation at 95°C for 2 minutes, 40 cycles of 95°C for 30 seconds, 46‐55°C for 30 seconds, and 72°C for 30 seconds. The human gene *GAPDH* was used as an internal control. The relative gene expression levels were analyzed by the 2^−∆∆Ct^ method. Three independent replicates of each experiment were performed.

**Table 1:** Sequences of the primer pairs used to analyze the genes

ID	Primer sequence (5′‐3′)	Tm (°C)	Ct
hsa_circ_0005505	R: GCCAGCTGCTTGAAAGTCTC F: TGCAGTGTAAGAAGCATTGGA	53	25‐32
hsa_circ_0001394	R: TCCATCAGTCATCTTGGTCCA F: TTCATGAAGCTGAGGAGGGG	55	25‐27
hsa_circ_0000798	R: GTACCTGCATCTGGGGTGAC F: ACTCCTGGACAAGGATCTGC	55	23‐25
hsa_circ_0004771	R: AGCTCACAATCCAAACACTTCC F: ACTTTTCAACAGCCTTCTCAAT	48	28‐32
hsa_circ_0001074	R: TTACTCTTTGATTTACGACTGC F: GTAACTCTGTCCTTATTATCGG	46	31‐35
hsa_circ_0067735	R: TCTCTGCACTCTTCACATTCCA F: TGACTTGTGCCTATTATTCTGC	48	26‐32
*GAPDH*	R: GGTCTGGGAGCCTGGAAAA F: TTCGCTCCTGGAAGATGGTAAT	46‐55	16‐24

Ct, cycle threshold; F, forward; R, reverse; Tm, annealing temperature.

### 2.6 Prediction of circRNA‐miRNA‐mRNA interactions

circRNA binding to a miRNA allows for its indirect regulation of the translation of an mRNA.^4^ We utilized miRanda v3.3a (http://www.microrna.org/microrna/home.do) and RNAhybrid 2.1 (https://bibiserv.cebitec.uni-bielefeld.de/rnahybrid/) as tools to predict the related target miRNAs and mRNAs for circ_0000798. Ultimately, the graphs of circRNA‐miRNA‐mRNA sharing meaningful correlation interaction networks were drawn using Cytoscape 3.3.0 (https://cytoscape.org/).

### 2.7 Statistical analysis

In this study, after tested by the one‐sample Kolmogorov‐Smirnov test (Table S1), the data in Figure 2a,b were analyzed by *t*‐test with Welch's correction in case of the normal distribution among groups; otherwise, the data were analyzed by Mann‐Whitney *U*‐test among groups (Table S2). The relationship between the differentially regulated circRNA and clinical features were analyzed using the chi‐square test. Survival analysis was carried out by Kaplan‐Meier analysis, and statistical analysis was performed through log‐rank test. A ROC curve was computed and the specificity and sensitivity of predictive power was assessed by the area under the curve (AUC) to indicate the diagnostic value of the selected dysregulated circRNA in the PBMCs of HCC patients compared to healthy controls. *P *<* *0.05 was considered statistically significant. All the data were analyzed using SPSS statistical software (version 17.0; SPSS, Chicago, IL, USA).

## 3 RESULTS

### 3.1 circRNA expression profiling in PBMCs from HCC patients and healthy controls

**Figure 1:**
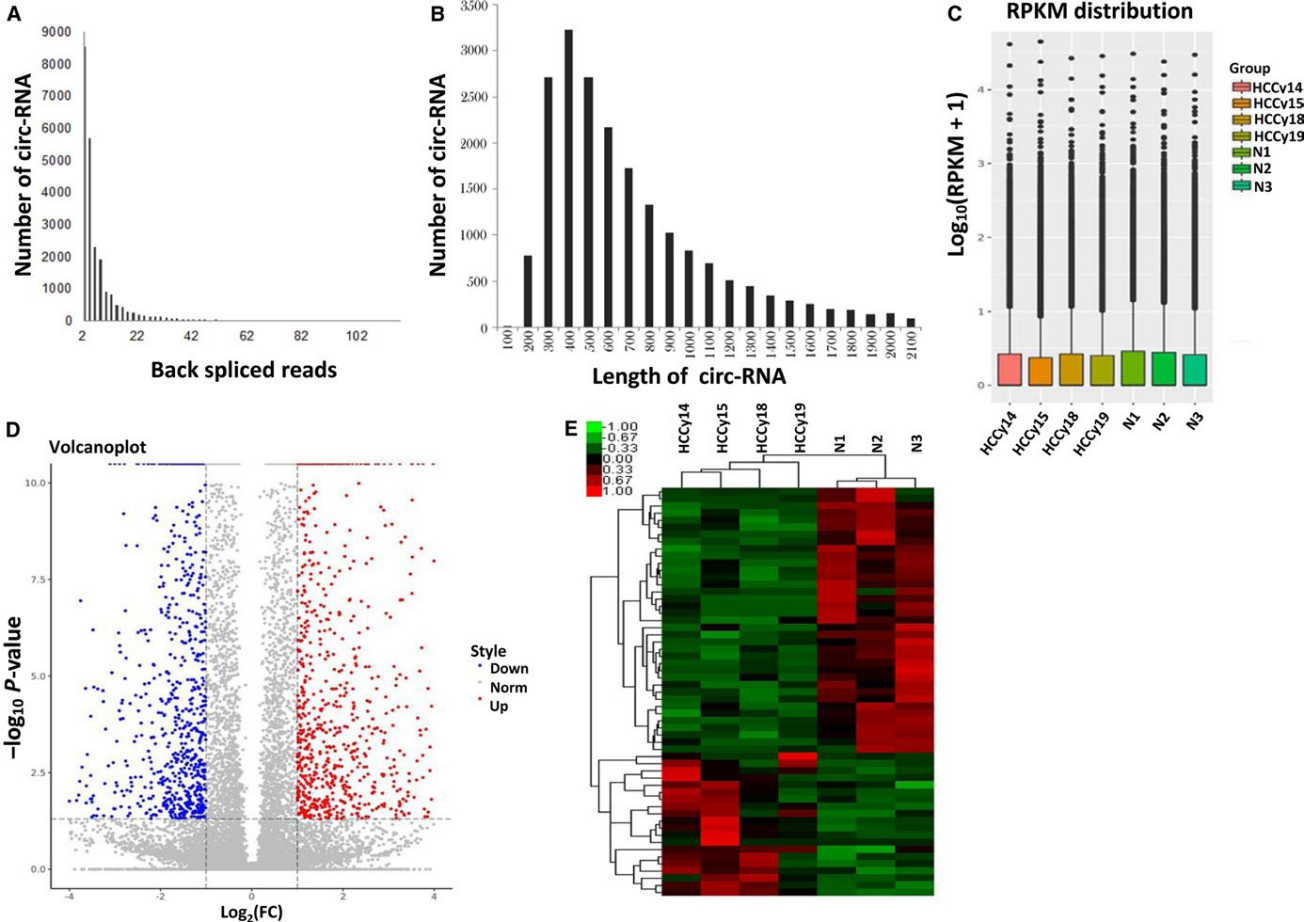
Dysregulated Circular RNA (circRNA) expression profiles in hepatocellular carcinoma (HCC) samples compared with normal samples from healthy controls. (a) The number of circRNAs in HCC samples compared with normal samples. The *Y* axis representing the circRNA supported reads number and the *X* axis representing the circRNA number with such reads supported were used to display the predicted circRNA and the circRNA prediction quality. (b) Length distribution of circRNAs. (c) A box plot showed the normalized intensities from the four HCC examples and three control examples. The box plots represent the interquartile range (25%‐75%, IQR) with whiskers corresponding to 1.5 × IQR and points—to outliers. (d) A volcano plot showing the dysregulated circRNA expression in the samples. Red and blue represent the up‐regulated and down‐regulated circRNAs in HCC, respectively. (e) Heat map of circRNAs showing hierarchical clustering of circRNAs with altered expression in HCC samples compared with normal samples. Each row represents one circRNA, and each column represents each sample. Cluster plot was done utilizing Cluster 3.0 software (open source) based on the normalized circRNA expression data using the parameter: 1. average linkage; 2. Pearson correlation. The up‐ and down‐regulated genes are colored in red and green, respectively. N1, N2, and N3 represent healthy controls, and HCCy15, HCCy19, HCCy14, and HCCy18 represent HCC patients

Based on the result of ACFS circRNA prediction and identification pipeline, the supporting reads for each circRNA was calculated, representing the confidence of the predicted circRNA. Global transcriptome expression analysis detected 5609 distinct circRNA candidates (Figure 1a). The size of the circRNAs ranged from 100 to 2100 nt (Figure 1b). Expression of the circRNAs in HCC patients and healthy controls was measured based on RPKM (mapped back‐splicing junction reads per million mapped reads), which demonstrated the normalized intensities from the seven samples (Figure 1c). The variations in circRNA expression were illustrated according to the volcano plot (Figure 1d). Hierarchical clustering heatmap analysis displayed a distinguishable circRNA expression profile among the samples (Figure 1e). These results showed that there was a markedly distinguishable circRNA expression profile in PBMCs between the HCC and healthy controls. Specifically, 58 circRNAs were significantly changed in the HCC patients compared with the healthy controls, of which 21 were up‐regulated and 37 were down‐regulated in the HCC group (Table S3). Significant differences in circRNAs between HCC patients and healthy controls were classified as fold change ≥2 or fold change ≤0.5, *P*‐value <0.05, and false discovery rate <0.05, as determined by having to satisfy both of the above conditions. The RNA‐seq data have been submitted to the NCBI Gene Expression Omnibus (GEO), under GEO accession number GSE120663 (https://www.ncbi.nlm.nih.gov/geo/query/acc.cgi?acc=GSE120663).

### 3.2 Validation of differentially expressed circRNAs by qRT‐PCR

**Figure 2:**
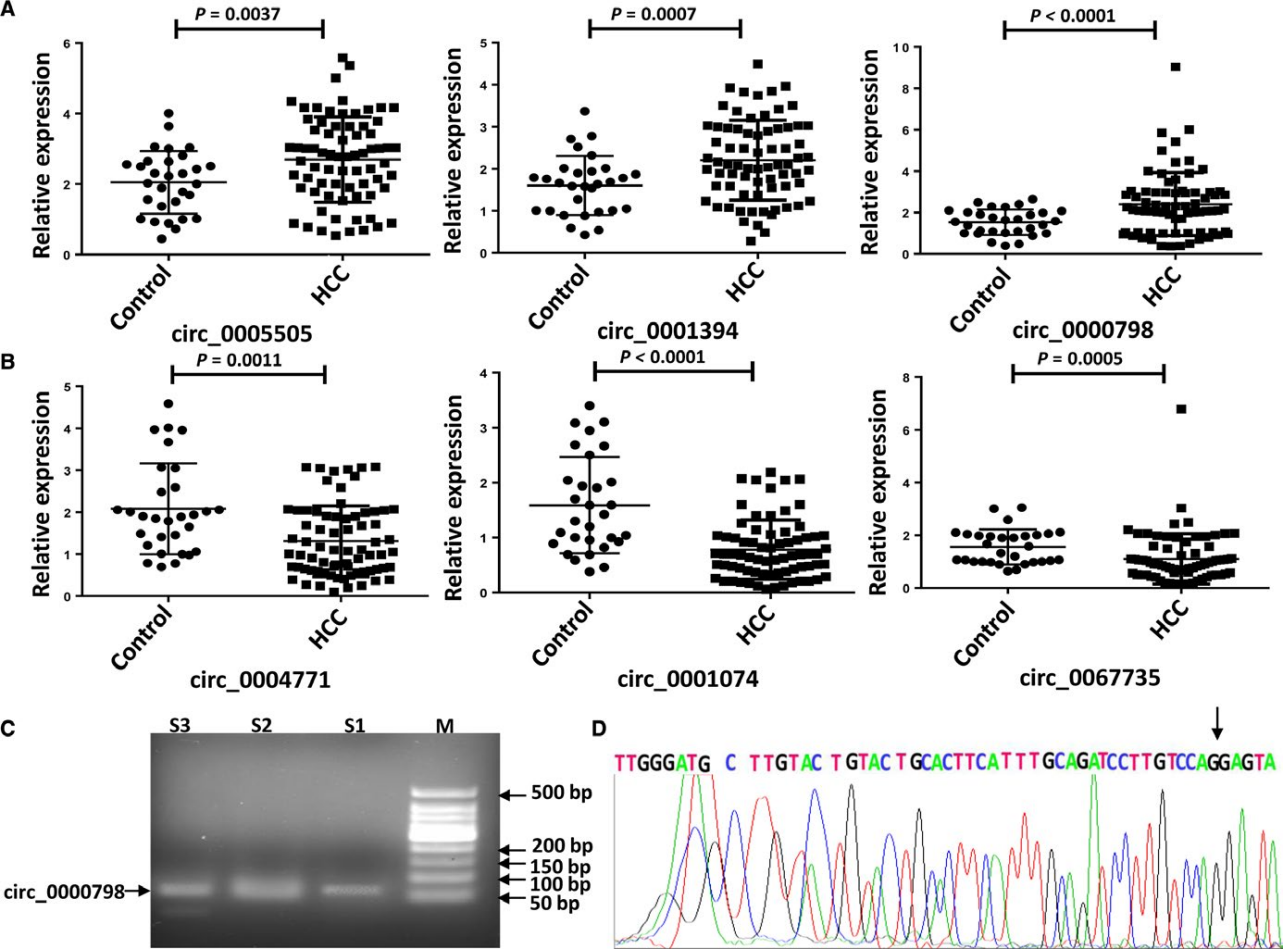
Validation of the expression of selected Circular RNAs (circRNAs) by qRT‐PCR. The relative expression of specific circRNAs between the peripheral blood mononuclear cells of hepatocellular carcinoma (HCC) patients and healthy individuals (controls). Relative expression of the three up‐regulated (a) and the three down‐regulated (b) circRNAs were determined by qRT‐PCR. The differences in the expression of circRNA candidates between HCC group and healthy group were analyzed by *t*‐test with Welch's correction, except circ_0067735, of which the expression difference was calculated by Mann‐Whitney *U*‐test. The data are presented as the means ± SD of three independent experiments. (c) The circRNA_0000798 expression was analyzed by qRT‐PCR, followed by 2.5% agarose gel electrophoresis. Lane M is the DL0501 Marker (GENERAY, Shanghai, China); Lanes S1‐S3 are the qRT‐PCR products of three repeated samples. (d) Validation of the circRNA_0000798 splicing junction site through sequencing of its qRT‐PCR product. The 2^−ΔΔCt^ method was used to calculate the circRNAs expression level relative to the *GAPDH* housekeeping control. Three independent replicates of each experiment were performed

To validate the sequencing data, six circRNAs, representing three of the up‐regulated circRNAs (circ_0005505, circ_0001394, and circRNA_0000798) and three of the down‐regulated circRNAs (circ_0004771, circ_0001074, and circRNA_0067735) (Table 2), were randomly selected from the 58 significantly dysregulated circRNAs (Table S1) for validation by qRT‐PCR using PBMCs of 72 HCC patients and 30 healthy controls. The results showed that the expression profiles of these six circRNAs were consistent with those from the sequencing results (Figure 2a,b). Among the six selected circRNAs, circRNA_0000798 showed the greatest stability among all subjects, as compared with the other five circRNAs (Figure 2a,b). Accordingly, the qRT‐PCR product of circRNA_0000798 was then subjected to agarose gel electrophoresis and Sanger sequencing, which demonstrated the expected 86‐bp size and correct sequence of the product (Figure 2c,d). Collectively, these data suggest the accuracy and reproducibility of the RNA sequencing findings.

**Table 2:** The six selected circRNAs in HCC samples compared with normal samples

circRNA ID	Log2FC	FDR	Regulation	Chr	Strand	Gene symbol	Genomic length, bp	Spliced length, bp
hsa_circ_0005505	1.172 122	0.019 256	Up	Chr12	+	*IRAK3*	24 660	754
hsa_circ_0000798	3.945 709	4.43E‐10	Up	Chr17	+	*BPTF*	2898	1226
hsa_circ_0001394	2.932 023	5.46E‐05	Up	Chr4	+	*TBC1D14*	739	739
hsa_circ_0001074	−4.708 075	0.004 433	Down	Chr2	−	*ORC4*	3237	242
hsa_circ_0004771	−2.045 652	5.71E‐13	Down	Chr21	−	*NRIP1*	29 231	203
hsa_circ_0067735	−1.838 783	0.003 161	Down	Chr3	+	*MED12L*	11 647	457

The six selected circRNAs from sequencing.

Chr, chromosome; circRNA, circular RNA; FC, fold change; FDR, false discovery rate; HCC, hepatocellular carcinoma.

### 3.3 circ_0000798 up‐regulation is associated with clinical features of HCC

**Figure 3:**
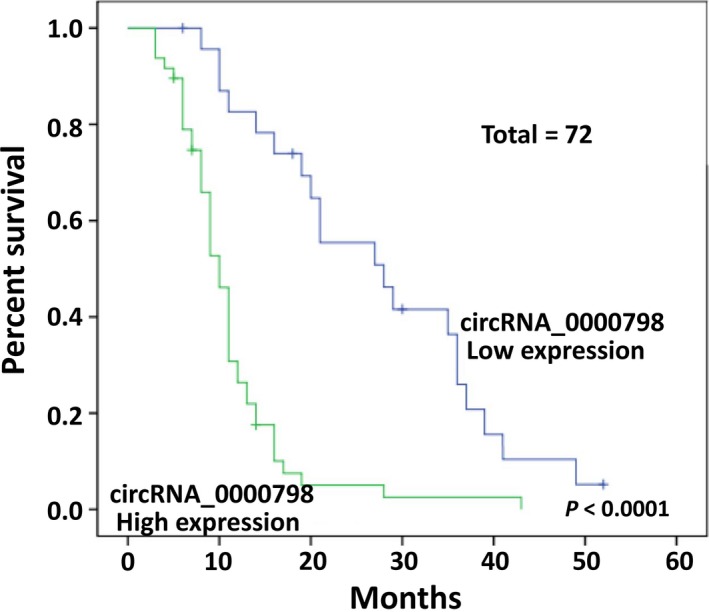
The correlation between circ_0000798 expression and hepatocellular carcinoma patients’ survival rate was analyzed using Kaplan‐Meier survival curves and log‐rank tests

We further selected a representative circRNA, circ_0000798, to investigate its clinical significance, because this circRNA was more stably up‐regulated among the 72 patients, as compared with the healthy controls (Figure 2a). The results demonstrated that there were no significant correlations between circ_0000798 expression in the PBMCs of HCC patients and the patients’ sex, age, family history, diabetes, α‐fetoprotein, tumor differentiation, lymph node status, and distant metastasis; however, circ_0000798 expression was markedly correlated with tumor size and cirrhosis (*P *<* *0.05) (Table 3). Moreover, to evaluate the prognostic significance of circ_0000798 expression in HCC, the HCC patients were divided into high‐ and low‐expression groups based on the expression levels of circ_0000798 detected in their PBMCs by qRT‐PCR. The results showed that HCC patients with high circ_0000798 expression level had a significantly lower survival rate than those with low circ_0000798 expression (median survival of 10 months vs 28 months; *P *<* *0.0001; Figure 3).

**Table 3:** The association between circ_0000798 expression and clinicopathological characteristics of 72 hepatocellular carcinoma patients

Characteristic	Total, n = 72	circ_0000798 expression		χ^2^	*P*
		Low, n = 24 (%)	High, n = 48 (%)		
Sex					
Male	16	7 (43.7)	9 (56.3)		
Female	56	17 (30.4)	39 (69.6)	1.004	0.316
Age, years					
<50	24	9 (37.5)	15 (62.5)		
≥50	48	15 (31.3)	33 (68.7)	0.281	0.596
Family history					
Yes	20	7 (35.0)	13 (65.0)		
No	52	17 (32.7)	35 (67.3)	0.035	0.852
Tumor size, cm					
<5	28	22 (78.6)	6 (21.4)		
≥5	44	2 (4.5)	42 (95.5)	42.195	**<0.0001** *
Tumor differentiation					
Well/moderate	56	16 (28.6)	40 (71.4)		
Poor	16	8 (50.0)	8 (50.0)	2.571	0.109
Diabetes					
Yes	8	2 (25.0)	6 (75.0)		
No	64	22 (34.4)	42 (65.6)	0.018	0.895
Cirrhosis					
Yes	42	3 (7.1)	39 (92.9)		
No	30	21 (70.0)	9 (30.0)	31.114	**<0.0001** *
AFP					
≤20	21	8 (38.1)	13 (61.9)		
>20	51	16 (31.4)	35 (68.6)	0.303	0.582
Lymph node status					
Yes	22	5 (22.7)	17 (77.3)		
No	50	19 (38.0)	31 (62.0)	1.604	0.205
Distant metastasis					
M0	41	17 (41.5)	24 (58.5)		
M1	31	7 (22.6)	24 (77.4)	2.832	0.092

AFP, α‐fetoprotein.

* Statistically significant (*P *< 0.05); the *P*‐value was from the χ^2^.

### 3.4 Diagnostic values of circ_0000798 for HCC patients

**Figure 4:**
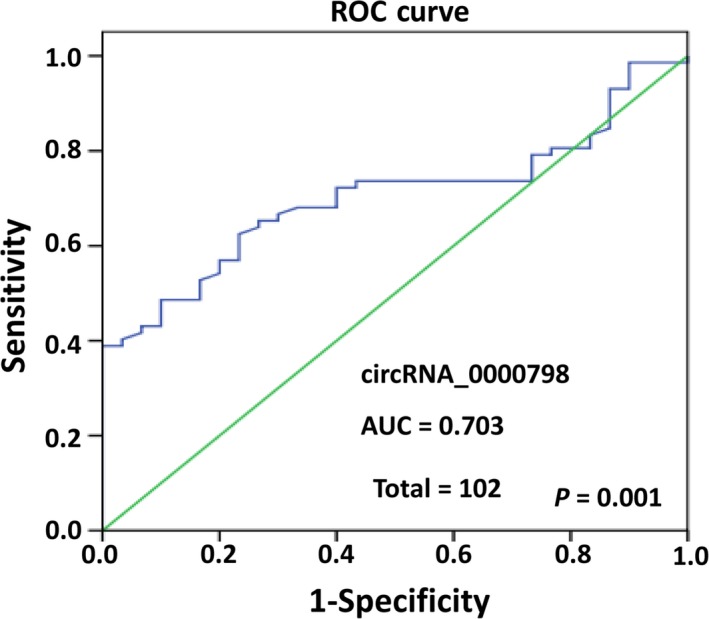
Analysis of the sensitivity and specificity of circ_0000798 as a novel hepatocellular carcinoma (HCC) marker by receiver operating characteristic (ROC) curve. The ROC curve was computed using the expression of fold changes of circ_0000798 in all samples to describe the circ_0000798 expression in discriminating HCC patients from healthy controls

To assess the potential value of significantly and differentially expressed circ_0000798 for HCC diagnosis, a ROC curve was computed to describe the circ_0000798 expression in discriminating HCC patients from healthy controls according the expression of fold changes of circ_0000798 in all samples. The results showed that the AUC of circ_0000798 reached 0.703 (95% confidence interval [CI]: 0.604‐0.803; *P* = 0.001), indicating that circ_0000798 in PBMCs could separate the patients with HCC from the healthy controls, and the circ_0000798 expression level might have potential clinical significance as a tumor marker (Figure 4).

### 3.5 Predicted circRNA‐miRNA‐mRNA regulatory network

**Figure 5:**
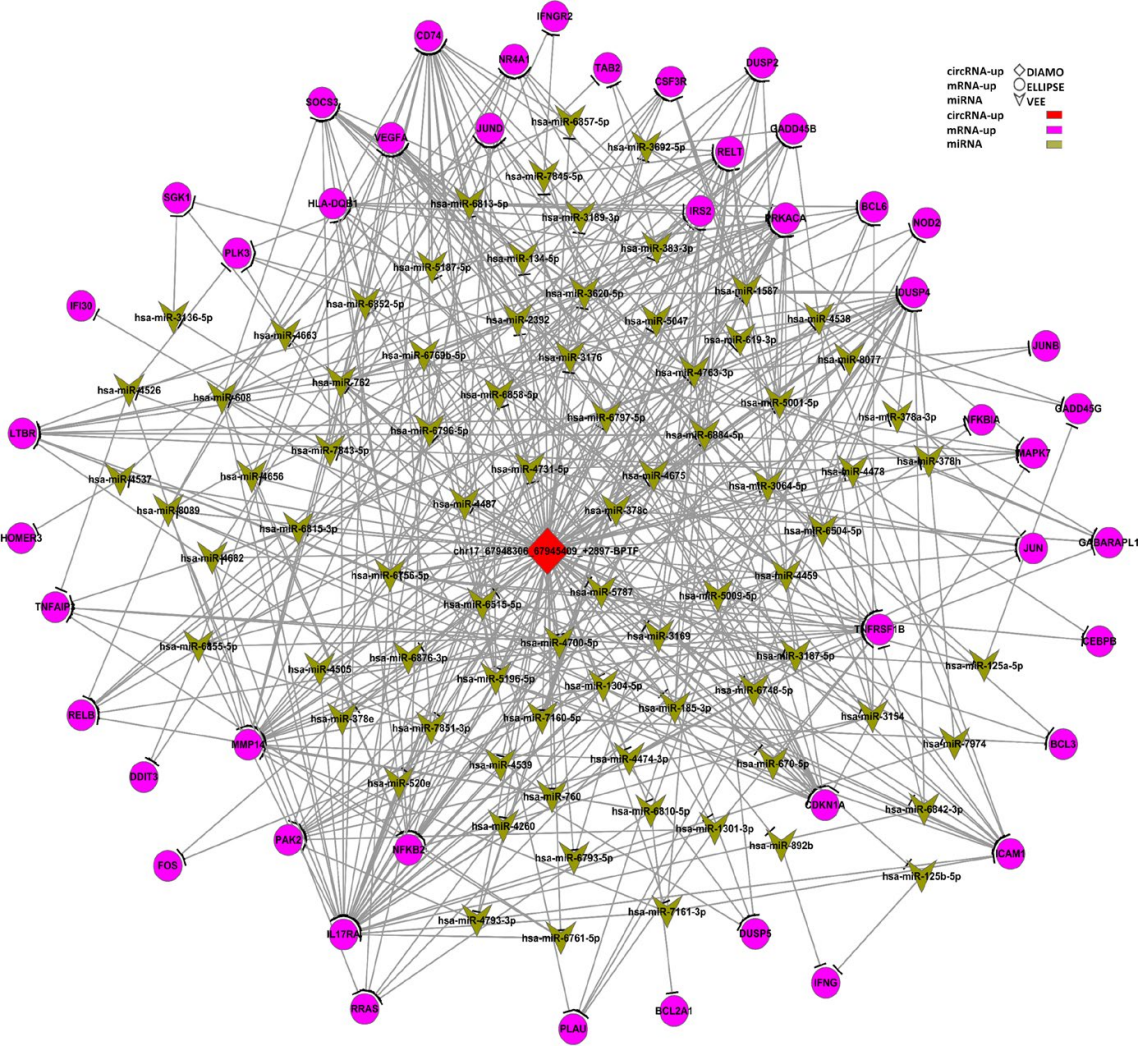
Competing endogenous RNA network in peripheral blood mononuclear cells from hepatocellular carcinoma

In order to further explore the potential functions of circ_0000798 in PBMCs of HCC patients, we performed a circRNA‐miRNA‐mRNA network analysis in which the immune‐related mRNAs from our RNA‐seq data were selected (Figure 5). We found that there were several potential target mRNAs of circ_0000798, mediated by the predicted miRNAs through “sponging” mechanisms. The predicted target mRNAs include the well‐known effector molecules of immune cells, such as *IFNG*,* IFNGR2*,* IL17RA*,* TNFAIP,* and *ICAM1*. In addition, the predicted target mRNAs also include the regulatory molecules in immune cells, mainly the transcription factors involved in the regulation of the proliferation, activation, and apoptosis, such as *FOS*,* BCL3*,* BCL2A1*,* BCL6*,* SOCS,* and *SGK1*.

## 4 DISCUSSION

Hepatocellular carcinoma is a high‐incidence malignant tumor, and most HCC patients are diagnosed at advanced stages of the disease, when there is tumor cell metastasis or diffusion. This advanced stage of disease at diagnosis causes a delay in treatment and supports poor prognosis.^19^ Recently, studies have found that circRNAs are important members of the ncRNA family in different species.^20^, ^21^ The development of high‐throughput sequencing technology has increased our understanding of the role and the molecular mechanism of circRNAs in human diseases, especially in cancer,^22^ and has promoted the development of these RNAs as a novel biomarker for cancer diagnosis.^23^, ^24^ In this study, circRNA sequencing was used to identify differences in the circRNA expression profile of PBMCs between HCC patients and healthy controls and has demonstrated the possible involvement of differentially expressed circRNAs in HCC pathology.

Compared with miRNAs and lncRNAs, circRNAs are widely expressed in human cells and more stable in mammalian cells,^9^ which endows circRNAs with the potential to be ideal biomarkers for human diseases. In the present study, to explore novel biomarkers of HCC, we first explored the expression profiles of circRNAs in PBMCs between four HCC patients and three healthy individuals (controls) by high‐throughput RNA sequencing. A total of 58 circRNAs were found to be significantly dysregulated (21 up‐regulated and 37 down‐regulated) in the HCC patients compared with the healthy controls. The expression of six randomly selected circRNAs (circ_0005505, circ_0001394, circRNA_0000798, circ_0004771, circ_0001074 and circRNA_0067735) were further analyzed by qRT‐PCR and confirmed as being consistent with the sequencing data.

Early diagnosis is the key to successful treatment and improved prognosis for HCC patients.^25^ circRNAs might be novel biomarkers, and have already been reported as helpful biomarkers for several diseases, including cancers such as gastric cancer, acute myeloid leukemia, pancreatic ductal adenocarcinoma, and lung cancer.^15^, ^16^, ^26^, ^27^ As such, they should be helpful for the diagnosis of HCC as well. Shang et al.^25^ found that circ_0005075 was up‐regulated in HCC tissues and its expression was positively related to the tumor size in HCC. In the current study, we found that several circRNAs were significantly up‐ or down‐regulated in PBMCs from HCC patients, among which circ_0000798 was selected for further investigation. As expected, circ_0000798 was confirmed as being markedly up‐regulated in the PBMCs of 72 HCC patients by qRT‐PCR. Prognostic analysis of circ_0000798 in these 72 HCC patients using Kaplan‐Meier analysis indicated that the up‐regulated circ_0000798 was always associated with larger tumor size and malignant cirrhosis. Furthermore, the survival rate of HCC patients with high circ_0000798 expression was significantly lower than those with low circ_0000798 expression. The AUC indicated that circ_0000798 had the potential to assist in the diagnosis for HCC. Together, these data indicate that circ_0000798 might be markedly correlated with poor prognosis in HCC.

Recently, many studies have indicated that circRNAs could function as a tumor regulator in HCC. For instance, Yu et al.^28^ showed that the expression level of Cdr1as was up‐regulated in HCC tissues, silencing of which could suppress the proliferation of HCC cells (SMMC‐7721 and HepG2). Qin et al.^12^, ^29^ found that down‐regulation of hsa_circ_0001649 in HCC cells significantly increased the mRNA level of matrix metallopeptidases 9, 10, and 13, promoting the metastasis of HCC. Zhu and colleagues^30^ demonstrated that the circRNA, circ_0067934, promotes tumor growth and metastasis in HCC through regulation of the miR‐1324/FZD5/Wnt/b‐catenin axis. All these findings strongly support the notion that circRNAs play important roles in tumor progression.

In our latest study, presented herein, we used RNA‐seq data to construct a circRNA‐miRNA‐mRNA network in order to predict the potential functions of circ_0000798 in PBMCs of HCC patients. We found that the potential target mRNAs of circ_0000798 include the well‐known effector molecules of immune cells, such as *IFNG*,* IFNGR2*,* IL17RA*,* TNFAIP,* and *ICAM1*. Moreover, the predicted target molecules also include molecules that have been verified to function in immune cells, such as *FOS*,* SOCS*,* SGK1,* and *BCL* family members. For instance, an earlier study found that after activation by mTORC2, *SGK1* promoted T helper type 2 differentiation by negatively regulating degradation of the transcription factor JunB mediated by the E3 ligase Nedd4‐2, and that mice with selective deletion of *SGK1* in T cells were resistant to experimentally induced asthma, as a result of a substantial IFN‐γ increase in response to viral infection and more readily rejected tumors.^31^ In addition, Li et al.^32^ reported that the mRNAs of *SOCS* family genes could be marked by m6A, increasing the levels of mRNAs and proteins of *SOCS* family members in Mettl3‐deficient naive T cells. The up‐regulated *SOCS* family activity subsequently inhibited IL‐7‐mediated *STAT5* activation and T‐cell homeostatic proliferation and differentiation.

## 5 CONCLUSION

This study is the first to assess circRNAs expression in PBMCs from HCC patients and compare the data with that from healthy controls. Bioinformatics analysis indicated that the up‐regulated expression of circ_0000798 was closely related to the development of HCC. Furthermore, circ_0000798 may be helpful for HCC diagnosis or acts as an independent prognostic biomarker for HCC patients. These observations and verifications have potential clinical significance, as dysregulated circRNAs such as circ_0000798 are potential diagnostic markers in HCC patients. In addition, bioinformatic analysis revealed that circ_0000798 could be involved in the pathophysiology of HCC by regulating the functions of peripheral immune cells and the consequent occurrence and progression of HCC. The predicted functions of this study's identified circ_0000798 in PBMCs of HCC patients should be further characterized with loss‐ and gain‐of‐function experiments in the future, which are expected to give further insight into how circ_0000798 exerts its functions on HCC by acting as a miRNA sponge.^33^

## CONFLICT OF INTEREST

The authors declare no conflicts of interest related to this publication.
