# Supplementary material for: Circular RNA expression profiles of peripheral blood mononuclear cells in hepatocellular carcinoma patients by sequence analysis
Source: Cancer Med. 2019 Feb 4;8(4):1423–33. doi: 10.1002/cam4.2010 (PMC6488130; doi:10.1002/cam4.2010)
Supplement: Supplementary file 1 [file CAM4-8-1423-s001.docx]

**Supplementary Table 1.** The test of index distribution

| CircRNA | Group | Number | $\bar{x}\pm SD$ | Statistical Z | *P* |
| --- | --- | --- | --- | --- | --- |
| circ_0005505 | HCC | 72 | 2.7006±1.20876 | 0.598 | 0.867 |
|  | Control | 30 | 2.0520±0.88941 | 0.605 | 0.858 |
| circ_0001394 | HCC | 72 | 2.2064±0.95223 | 0.613 | 0.846 |
|  | Control | 30 | 1.6040±0.70098 | 0.650 | 0.792 |
| circ_0000798 | HCC | 72 | 2.4055±1.52644 | 1.323 | 0.060 |
|  | Control | 30 | 1.5283±0.61700 | 0.617 | 0.842 |
| circ_0004771 | HCC | 72 | 1.3080±0.84654 | 1.138 | 0.150 |
|  | Control | 30 | 2.0812±1.08599 | 1.142 | 0.147 |
| circ_0001074 | HCC | 72 | 0.7819±0.53962 | 1.157 | 0.137 |
|  | Control | 30 | 1.5882±0.87616 | 0.814 | 0.522 |
| circ_0067735 | HCC | 72 | 1.1069±0.95546 | 1.718 | 0.005 |
|  | Control | 30 | 1.5582±0.66301 | 1.115 | 0.167 |
