# Supplementary material for: Circular RNA expression profiles of peripheral blood mononuclear cells in hepatocellular carcinoma patients by sequence analysis
Source: Cancer Med. 2019 Feb 4;8(4):1423–33. doi: 10.1002/cam4.2010 (PMC6488130; doi:10.1002/cam4.2010)
Supplement: Supplementary file 2 [file CAM4-8-1423-s002.docx]

**Supplementary Table 2.** The statistical results of the six circRNAs expression between HCC group and control group

| CircRNA | Group | Number | Statistical (t or U) | *P* |
| --- | --- | --- | --- | --- |
| circ_0005505 | HCC | 72 | **t** value | 0.0013 |
|  | Control | 30 |  |  |
| circ_0001394 | HCC | 72 | **t** value | 0.0007 |
|  | Control | 30 |  |  |
| circ_0000798 | HCC | 72 | **t** value | <0.0001 |
|  | Control | 30 |  |  |
| circ_0004771 | HCC | 72 | **t** value | 0.0002 |
|  | Control | 30 |  |  |
| circ_0001074 | HCC | 72 | **t** value | <0.0001 |
|  | Control | 30 |  |  |
| circ_0067735 | HCC | 72 | **U** value | 0.0005 |
|  | Control | 30 |  |  |
